# ERP Correlates of Proactive and Reactive Cognitive Control in Treatment-Naïve Adult ADHD

**DOI:** 10.1371/journal.pone.0159833

**Published:** 2016-07-22

**Authors:** Venke Arntsberg Grane, Jan Ferenc Brunner, Tor Endestad, Ida Emilia S. Aasen, Juri Kropotov, Robert Thomas Knight, Anne-Kristin Solbakk

**Affiliations:** 1 Department of Neuropsychology, Helgeland Hospital, Mosjøen, Norway; 2 Department of Psychology, University of Oslo, Oslo, Norway; 3 Department of Physical Medicine and Rehabilitation, Trondheim University hospital – St.Olav, Trondheim, Norway; 4 Department of Psychology, Norwegian University of Science and Technology, Trondheim, Norway; 5 Institute of Human Brain, Russian Academy of Science, St. Petersburg, Russian Federation; 6 Helen Wills Neuroscience Institute and Department of Psychology, University of California, Berkeley, United States of America; 7 Department of Neurosurgery, Division of Surgery and Clinical Neuroscience, Oslo University Hospital – Rikshospitalet, Oslo, Norway; Harvard Medical School/Massachusetts General Hospital, UNITED STATES

## Abstract

This study investigated whether treatment naïve adults with Attention Deficit Hyperactivity Disorder (ADHD; n = 33; 19 female) differed from healthy controls (n = 31; 17 female) in behavioral performance, event-related potential (ERP) indices of preparatory attention (CueP3 and late CNV), and reactive response control (Go P3, NoGo N2, and NoGo P3) derived from a visual cued Go/NoGo task. On several critical measures, Cue P3, late CNV, and NoGo N2, there were no significant differences between the groups. This indicated normal preparatory processes and conflict monitoring in ADHD patients. However, the patients had attenuated Go P3 and NoGoP3 amplitudes relative to controls, suggesting reduced allocation of attentional resources to processes involved in response control. The patients also had a higher rate of Go signal omission errors, but no other performance decrements compared with controls. Reduced Go P3 and NoGo P3 amplitudes were associated with poorer task performance, particularly in the ADHD group. Notably, the ERPs were not associated with self-reported mood or anxiety. The results provide electrophysiological evidence for reduced effortful engagement of attentional resources to both Go and NoGo signals when reactive response control is needed. The absence of group differences in ERP components indexing proactive control points to impairments in specific aspects of cognitive processes in an untreated adult ADHD cohort. The associations between ERPs and task performance provided additional support for the altered electrophysiological responses.

## Introduction

Attention Deficit Hyperactivity Disorder (ADHD) is a prevalent early-onset, neurodevelopmental syndrome characterized by severe and developmentally inappropriate levels of inattention, hyperactivity, and impulsivity (DSM V—American Psychiatric Association, Diagnostic and Statistical Manual of Mental Disorders, 2013). The core behavioral features of ADHD persist into adulthood in a substantial number of individuals and are associated with significant social, academic, vocational, and financial burden [1–7].

### Neuropsychology of cognitive control in ADHD

Neuropsychological studies report reduced performance in a variety of cognitive tasks, including tasks demanding selective and sustained attention, motor response inhibition, working memory, and timing control, as well as more behaviorally complex executive functions such as planning and decision-making [8–11]. A common denominator of most cognitive tasks examined is the demand for voluntary control over attentional focus and appropriate monitoring of behavioral output. Such cognitive or executive control is linked with the prefrontal cortex (PFC) and its connections with subcortical and parietal network nodes [12–14].

Findings from functional neuroimaging studies have revealed altered hemodynamic response patterns in fronto-striatal and fronto-parietal circuits of individuals with ADHD during both resting state and cognitive tasks [13, 15, 16], including tasks demanding inhibitory motor control [17, 18]. One of the most influential theoretical models of ADHD postulates that a basic deficit in inhibitory control is at the core of the disorder [8, 19–22], and underlies the diversity of executive difficulties observed in ADHD. The inhibitory control hypothesis offers a parsimonious but simplistic account of the neurobiology of ADHD [23–26]. A related model that includes aspects of Barkley’s inhibition model and other influential models [27–31] is the behavioral neuroenergetics model of ADHD [32]. Briefly, this theory posits that impaired task performance, particularly inconsistent and slow responding can be attributed to ‘energetic’ insufficiency of neural networks [32].

The capacities to allocate attentional resources, anticipate and prepare for upcoming events, and suppress inappropriate responses are critical cognitive control functions that are prerequisites for complex goal-directed behavior. Deficits in any of these basic control functions may contribute to difficulties with flexible and timely behavioral adjustment to changing environmental demands, as is typically seen in individuals with ADHD [33–35].

An inherent limitation of behavioral methods for assessing covert cognitive control functions, such as preparation for- or inhibition of action, is that the rapid neural processes subserving them can only be indirectly inferred from the presence or absence of behavioral responses. However, stages of neural information flow evoked by an event can be captured with millisecond time resolution by examining event-related brain potentials (ERPs) extracted from ongoing electroencephalographic (EEG) activity recorded from the scalp. ERPs have been particularly successful for understanding the neural basis of attentional processes [9, 10, 35–43], and provide a means to characterize the neurophysiological basis of the core symptoms of ADHD [44, 45]. The ERP method has been extensively employed in studies of childhood ADHD [35, 44, 46–49], and in recent years, ERP studies have given an increasing focus also on adult ADHD [50–56]. The objective of the current study was to examine electrophysiological indices of control processes related to both anticipatory attention and preparation for action, responses election to subsequent targets, and how these processes might be affected in adult ADHD.

### ERPs in cued Go/NoGo paradigms as indices of proactive and reactive attentional control

In a cued Go/NoGo task, a defined cue (S1) signals that the subsequent stimulus (S2: Go or NoGo) *may* require a response. When cues are mostly valid signals for Go stimuli, they evoke increased motor preparation processes facilitating speeded reactions. However, preparation for fast responding leads to an augmented need for abortion of the prepared response whenever a NoGo stimulus occurs [39]. Thus, the cued Go/NoGo task requires both proactive and reactive control processes. Reactive control processes are correction mechanisms (i.e., response suppression or alternative response facilitation) initiated *after* detection of errors or high interference events such as NoGo signals. Proactive control, in contrast is triggered by contextual cues occurring *in advance of* goal-relevant events, and convey information to promote appropriate behavioral responses [57].

An electrophysiological correlate of attentional preparatory processes is a broad positive-polarity deflection starting about 300 ms after cue (S1) onset. It is denoted Cue P3andhasa maximum over centro-parietal electrode sites [58, 59]. It is proposed to reflect allocation of attentional resources for expected targets [60–62], stimulus relevance evaluation, and activation of the response rules related to the imperative stimulus categories [63, 64]. The Cue P3 is followed by a sustained negative-polarity shift in the EEG known as the contingent negative variation (CNV) [65] that is typically measured at the fronto-parietal midline electrode sites. The CNV typically starts in the late part of the time window between the cue (S1) and lasts until the second stimulus of the S1-S2 pair appears [66, 67]. The CNV is generally measured at fronto-parietal midline electrode sites. This PFC-mediated preparatory control exerts top-down influence on sensory and motor cortical areas [66–68].The CNV is thought to index motor preparation and anticipatory attention for the upcoming imperative stimulus [69]. Its late phase has been suggested to reflect sustained attention to task-set representation over time [37].

The presentation of an S2, defined as the target, generates positive-polarityGoP3 potentials that have partially overlapping central-parietal distributions [70]. The amplitude of the Go P3 has been proposed to reflect the allocation of attentional resources [42, 71], and activation of an event-categorization network thought to be controlled by the joint operation of attention and working memory [72]. The centrally distributed Go P3 has been interpreted to index a stimulus evaluation and classification process [73], whereas the parietally maximum Go P3 has been linked to response selection [73, 74].

Cancellation of the prepared response is required when S2 occasionally constitutes a NoGo signal. The NoGo signal evokes two succeeding ERPs called NoGo N2 and NoGo P3. Both have a fronto-central distribution, where NoGo N2 has a pronounced negativity 200–300 ms after the onset of S2 [75, 76], and NoGo P3 peaks after approximately 340 ms [39, 77, 78]. The NoGo N2 is thought to reflect both perceptual and cognitive competition between the Go and the alternative NoGo response [79–83], and to represent attentional mismatch processes[84], or conflict related monitoring [39].

The NoGo P3 [39, 85, 86] has recently been proposed to index evaluation of the inhibitory process or its outcome rather than the actual motor inhibition [39, 87]. Using independent component analysis, Brunner and colleagues (2015) found that the NoGo P3 consists of at least two components, which may account for both of the suggested processes. They proposed that one may reflect the process of replacement of a prepared Go-response with an alternative response, while the later component may reflect monitoring of these preceding processes [37].

Regardless of the exact functional meaning of the ERP components generated to the cued Go/NoGo task, they can be viewed as indices of proactive (Cue P3, late CNV) and reactive (Go P3, NoGo N2, and NoGo P3) cognitive control processes. The present study aimed at examining ERP markers of proactive and reactive attentional control in unmedicated adult ADHD patients.

### ERP correlates of attentional control in ADHD

Despite the focus on altered P3 as a possible endophenotype for childhood ADHD [88] there are still limited ERP studies on adults and even fewer on treatment-naive adults with ADHD. A meta-analysis by Szuromi and colleagues included 6 adult ADHD studies and found an overall decrease in P3 amplitude during target detection [45]. Some studies have focused on NoGo N2 and/or P3 components as neurophysiological indices of motor response inhibition deficits in ADHD [52–54, 56, 89–91]. There is, however, electrophysiological evidence that deficits in attentional orienting and motor preparation may precede or accompany deficits in reactive motor response processes [23, 36, 60, 89, 92, 93].

Using variants of cued Go/NoGo tasks, the few studies of adult ADHD found reduced P3 to cues [89], and the ensuing CNV [89, 90, 93], as well as attenuated fronto-central P3 to NoGo stimuli [89, 90, 94, 95]. The similarity of these ERP findings from cross-sectional studies to those reported in the childhood ADHD literature [60, 93, 96–98] supports persistence of the neurocognitive processing deficits into adulthood. A longitudinal study by Doehnert and colleagues found the NoGo P3 to develop later and the NoGo P3, Cue P3, and CNV to remain reduced in the ADHD group compared to healthy controls when measured from childhood to adolescence [60]. Examining the course of electrophysiological indices of preparation (Cue P3, CNV) and response control (NoGo P3) in a cued Go/NoGo task in much the same small sample of ADHD patients (11 ADHD patients and 12 controls) the research group found that only the CNV remained attenuated until young adulthood [44]. The authors concluded that attenuated CNV appears to be a particularly robust and developmentally stable marker of ADHD and may serve as an endophenotype of the disorder. Although the findings are suggestive, we note that the study may be underpowered to detect other possible attenuated ERPs due to the small sample sizes. Another study, with 6 years follow-up data from 110 young people with childhood ADHD and 169 controls found that those with persistent symptoms differed from those with remitting symptoms with regard to smaller CNV amplitude and a similar non-significant pattern for Cue P3. When controlling for the lower IQ in the ADHD group with persistent symptoms, the difference in CNV amplitude was no longer significant, while the effect sizes remained similar for cue P3 amplitude [52]. Altogether, the studies provide support to the idea that the persistence of ADHD into adulthood may be related to deficient neural maturation.

### Aims and hypotheses of the present study

This study is unique in that we report data from ADHD patients with clinically significant difficulties who were examined prior to initiation of pharmacological treatment (e.g., methylphenidate or atomoxetine). This avoids the effects on brain function of any prior pharmacological treatment [99, 100]. Accordingly, the primary objective was to investigate ERP indices of preparatory and reactive attentional control in newly diagnosed unmedicated adult ADHD patients. We used a complex version of a visual cued Go/NoGo task requirering prioritizing of attentional resources and preparedness, as well as motor responses to Go stimuli and response withholding to NoGo stimuli. This task distinguishes children with ADHD from healthy control children on performance and spectral EEG parameters [101] and provides predictive information about acute side-effects of stimulant medication in pediatric ADHD [47]. Because the task elicits ERP components with high test-retest reliability in healthy adults [102] we chose to focus our analyses on known correlates of cognitive control such as the Cue P3 and the late CNV as indices of proactive processes, and the Go P3, as well as NoGo N2 and NoGo P3as indices of reactive control processes.

Based on the previous ERP literature on childhood ADHD, and the limited literature on adult ADHD, we proposed the following main hypotheses. The amplitudes of the Cue P3 and late CNV would be attenuated in the ADHD group relative to the healthy age-matched control group. We further predicted that the P3 to Go signals, and the N2 and P3 to NoGo signals would be diminished in the ADHD group. Behavioral concomitants of the electrophysiological parameters were also examined. Increased reaction time variability to Go signals is one of the most stable behavioral markers of ADHD in both childhood [24, 103] and adult ADHD [104, 105]. Accordingly, we anticipated that increased reaction time variability to Go stimuli might also characterize our adult ADHD cohort.

Because cognitive ERPs can be impaired in clinical disorders involving depression [106–108], anxiety [108], alcohol abuse [109], as well as antisocial personality problems [110], and such problems frequently coexist with adult ADHD symptoms [5],we correlated the electrophysiological measures with self-rated scores on the Depression, Anxiety and Antisocial Personality Problems scales derived from the Achenbach System of Empirically Based Assessment (ASEBA) Adult Self-Report (ASR).

## Materials and methods

### Participants

The initial study samples had 36 ADHD patients and 35 healthy controls. However, 2 patients and 4 controls had excessive noise (mainly movement artifacts) in the electrophysiological recordings and/or lack of behavioral response registration and were therefore excluded from the study. In addition, 1 ADHD patient was excluded due to excessive commission errors (6 times that of the patient with the next highest error rate). In the following, we report the results of a group of 33 treatment-naive adults with newly diagnosed ADHD (14 male; mean age = 31.3 years; age range = 19–51 years; mean education = 11.5 years, education range = 10–16 years), and a comparison group of 31 healthy controls (14 male; mean age = 31.9; age range = 18–51 years; mean education = 12.5; education range = 10–17 years). The ADHD participants were recruited from a consecutive series of patients referred to the Department of Neuropsychology, Helgeland Hospital in the time period from 2008 to 2011. All patients were referred from specialist mental health services or primary care physicians for a second opinion evaluation of suspected ADHD. None had received stimulant medications prior to or during participation in the study, primarily because the ADHD diagnosis was not confirmed. Three patients with ADHD were treated with antidepressant medication at the time of study. All had taken the prescribed dose on the day before- but not on the day of assessment. Inclusion in the ADHD group was based on the DSM-IV criteria for ADHD [111] assessed by a senior neuropsychologist during a semi-structured interview for adults (Adult Interview) [112]. The interview comprises assessment of childhood and current DSM-IV-defined ADHD symptoms related to impairment or history of problems at school, psychiatric history, everyday functioning, as well as past and present comorbidities. Out of the 42 initially referred patients, 6 were not included in the study because they did not fulfill the DSM-IV diagnostic criteria for ADHD, and/or were rejected based on the other exclusion criteria. All patients included met the DSM-IV criteria for ADHD, combined type, as they had significant behavioral impairment related to inattention, hyperactivity, and impulsivity.

Healthy controls were recruited from three medium-size companies in the Helgeland area. Controls were selected based on the matching criteria sex, age, and years of education. From lists of all employees in the companies, those who fulfilled the matching criteria were asked to participate. In cases where more than one employee met the matching criteria for a patient, a random draw was conducted. In addition, healthy control participants underwent the diagnostic assessment. None fulfilled the DSM-IV diagnostic criteria for ADHD.

All participants were questioned about their past and present somatic and psychological health. Criteria for exclusion were: a) history of mild to severe traumatic brain injury, b) neurological disorder, c) history of severe memory problems, d) diabetes, e) metabolic disorder, f) psychotic symptoms, g) alcohol and/or substance abuse requiring treatment, and h) visual or auditory sensory loss. Prior to inclusion in the study, all participants were tested for auditory (audiometry test) and vision (optometry test) deficits. Participants had either normal acuity or vision corrected by optical lenses. None had impaired hearing. The participants were right-handed except for 1 in each group who was left-handed.

The participants were further evaluated for general IQ with the Wechsler Adult Intelligence Scale (WAIS-III) [113]. The patients and healthy controls did not differ significantly in age, sex, years of education, or IQ. The Adult Self-Report (ASR) of the Achenbach System of Empirically Based Assessment (ASEBA) was completed to attain an independent ADHD score, as well as information about neuropsychiatric comorbidity, alcohol use, and work/study status [114] (Table 1). All participants gave written informed consent and the study was approved by the Regional Committee for Medical and Health Research Ethics—North Norway (REC North), document number 2010/3361-6.

**Table 1:** Demographics, IQ scores on WAIS-III, and T-scores on the ASEBA Adult self-Report (ASR) DSM scales for ADHD patients (n = 33) and healthy controls (n = 31).

	ADHD		Control		
	Mean	SD	Mean	SD	p
**Age** (years)	31,3	9,6	31.9	10.0	.788
**Education** (years)	10,8	1,6	11,3	2,2	.057
**Gender** (M/F)	14/19		14/17		.825
**Work or study** (number/%)	19/58		31/100		.001
**WAIS-III** (IQpoints)					
FSIQ	93.7	13.0	98.9		.086
VCI _†_	93.3		95.6		.470
POI _†_	104.4		108.9		.200
WMI _†_	87.9		93.9		.081
PSI _†_	90.4		100.3		.001
**ASR DSM and Problem scales** (t-scores)					
ADHD _#_	74.7		52.2		.001
Depression _#_	67.1		50.9		.001
Anxiety _#_	59.2		50.4	0.9	.001
Antisocialβ	63.1	42469	52.0	42463	.001
Alcohol _use_ _π_	56,9	7,0	54.8	4,7	.220

Notes. Education = total years of schooling completed; Work or study = working or studying during the last 6 months; FSIQ = Full Scale Intelligence Quotient; VCI = Verbal Comprehension Index; POI = Perceptual Organization Index; WMI = Working Memory Index; PSI = Processing Speed Index; Alcohol use = days being drunk during the last 6 months;

^†^ = missing data in 1 ADHD patient;

^#^ = missing data in 1 ADHD patient and 1 control;

^β^ = missing data in 5 ADHD patients and 5 controls;

^π^ = missing data in 8 ADHD patients and 7 controls.

### Experimental task, EEG recording and analysis

#### Experimental task

The cued Go/NoGo task consisted of three categories of visual stimuli: 20 different images of animals, 20 different images of plants, and 20 different images of humans. The centrally presented stimuli subtended an approximate visual angle of 3°.

In order to maintain alertness and arousal in a long-lasting repetitive task [40, 115, 116] novel and irrelevant stimuli were presented in the fourth condition Plant—Human (both pictures of humans and a sound consisting of five 20 ms tones of different frequencies: 500, 1000, 1500, 2000, and 2500 Hz). Each time, a new combination of tones was used; the sounds were presented via two loudspeakers at an intensity of 70 dB SPL. The trials consisted of 4 different categories of stimulus pairs (S1-S2): animal-animal (A-A) pairs were Go trials, animal-plant (A-P) pairs were NoGo trials, plant-plant (P-P) pairs and plant-human (P-H) pairs were designated Irrelevant trials. The S1 and the S2 in animal-animal and plant-plant pairs were always identical pictures. Each stimulus of the pair was presented for 100 ms with an inter-stimulus interval of 1000 ms (SOA = 1100 ms), and forthcoming trials were separated by 1500 ms. Four hundred trials were grouped into 4 blocks where each block represented a unique set of five animal, five plant, and five human stimuli presented with equal probabilities for each trial category. Equal numbers of Go and NoGo trials are not expected to establish a strong prepotent Go response as in a typical paradigm where Go signals are more frequent than NoGo signals. However, electrophysiological results from studies using this paradigm support that differential responses were indeed evoked by the two stimulus classes [50, 117, 118].

**Fig 1:**
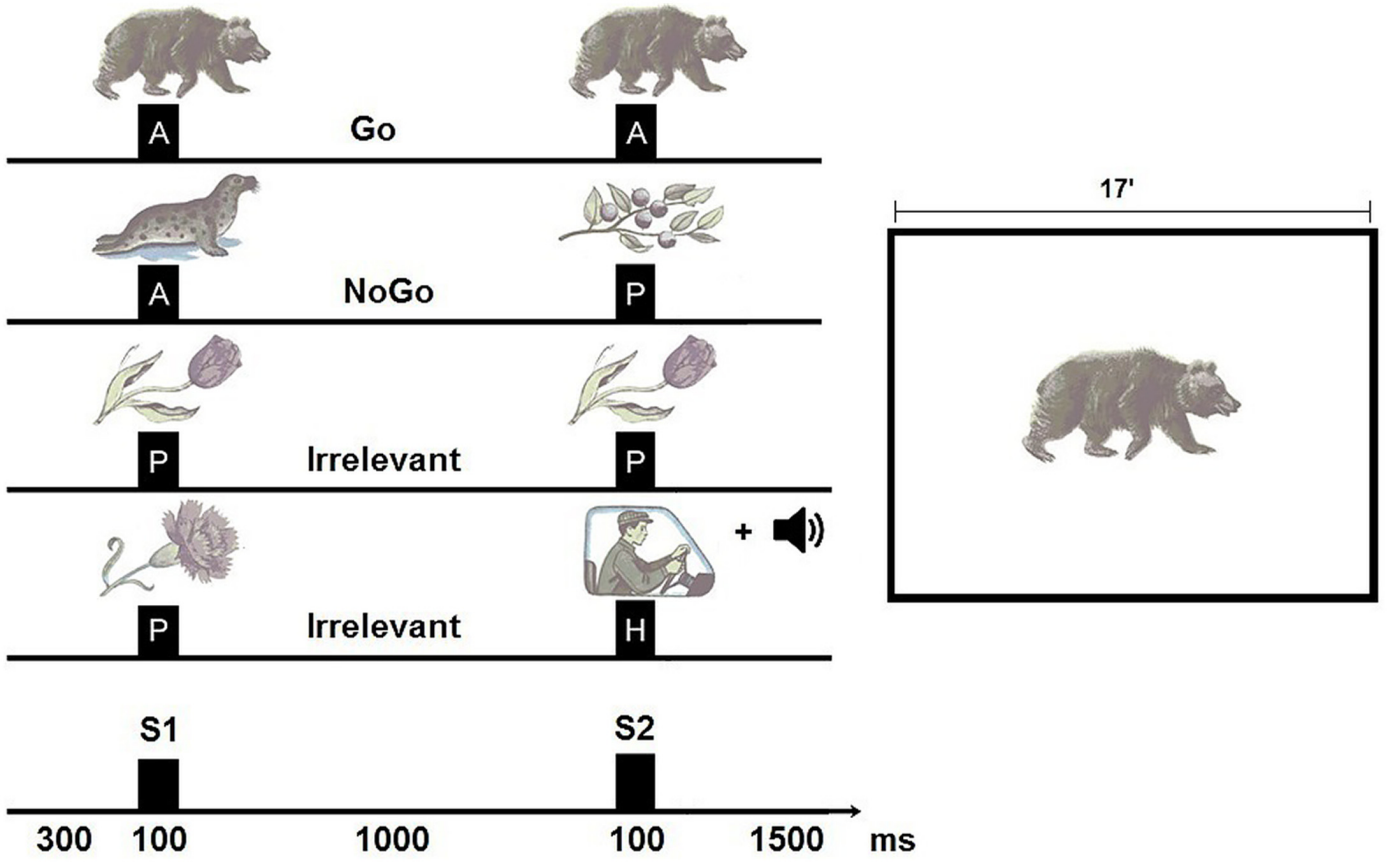
The cued Go/NoGo task. Left: The task was to respond to S2 in all animal-animal pairs (A-A) and withhold responses to S2 in all animal-plant pairs (A-P) and Irrelevant pairs (P-P, P-H). Each trial had a total duration of 3000 ms, where the first S1 and S2 had their onsets at 300 ms and 1400 ms into the trials, respectively. Duration of stimulus presentation was 100 ms. Right: Shows the size of the stimuli relative to the 17-inch computer screen.

The participants were instructed to press a button with their dominant index finger to S2 in all Go trials. They were told not to press the button to S2 in NoGo trials and Irrelevant trials. The total duration of the task was approximately 20 minutes with a 2 minutes break mid-way when 200 trials had elapsed (Fig 1). In the break, they were told that they had seen 200 picture pairs, and that additional pictures would be presented after the break. The experimental task differed from traditional continuous performance tasks (CPTs) with regard to the complexity of stimuli and conditions, and the mid-way break.

Omission errors, defined as not responding to S2 in Go trials, and commission errors, defined as responding to S2 in NoGo trials, were calculated separately for each participant across trials. A button press was defined as a response and recorded if it occurred in the time interval from 200 ms to 1000 ms after presentation ofS2. Mean reaction time (RT) and standard deviation of reaction time (RT SD) were calculated across trials for each participant.

Event-related potentials (ERP): Electroencephalogram (EEG) was recorded using a Mitsar (www.mitsar-medical.com) 19-channel EEG system. Electrodes were placed according to the International 10–20 system, using an electrode cap with tin-electrodes (Electro-cap International Inc.). Impedance was kept below 5 kOhm. The input signals referenced to the linked ears were filtered between 0.3–50 Hz, and digitalized at a sampling rate of 250 Hz. EEG data were computed using Win EEG software (www.mitsar-medical.com) in common average montage. ERPs for each individual were computed for Go, NoGo and Irrelevant trials without errors. Error trials were automatically excluded from averaging. Eye blink artifacts were corrected by zeroing the activation curves of individual independent components corresponding to eye blinks. Epochs of the filtered EEG with excessive absolute amplitude (>100 uV), and/or slow (> 50 uV in the 0 to 1 Hz band), and excessive fast (> 35 uV in the 20 to 35 Hz band) frequency activity were automatically excluded from further analysis.

#### Extracting averaged ERPs and measurement of mean amplitudes

The ERP components were scored as mean amplitude at electrodes where the components of interest are known to be clearly present to reduce noise and increase the statistical power [115]. The Cue P3 was measured 300–550 ms after the presentation of the cue stimulus (animal) and the irrelevant stimulus (plant) at the Cz and Pz electrodes. The late CNV was measured in the last 100 ms before S2 at the midline electrodes Fz, Cz, and Pz in both relevant and irrelevant conditions. The mean number of artifact-free trials used to compute ERPs for the Cue P3 and the late CNV were 178.1 (SD = 24.1; range = 87–200) for the ADHD group, and 180.7 (SD = 23.1; range = 87–200) for the healthy control group. The mean number of artifact-free trials used to compute ERPs for the Cue P3 and late CNV in the Irrelevant conditions was 178.2 (SD = 25.4; range = 81–200) for the ADHD group, and 177.3 (SD = 25.3; range = 90–199) for the healthy control group.

The Go P3 was measured 280–380 ms after the onset of S2 in the Go condition at the Cz and Pz electrodes. The mean number of artifact-free trials used to compute the Go P3 was 83.3 (SD = 15.0; range = 43–100) for the ADHD group, and 89.7 (SD = 13.3; range = 33–99) for the healthy control group. In the NoGo condition, the NoGo N2 was measured at the Fz and Cz electrodes, 200–300 ms after the presentation of S2, and the NoGo P3 was measured in the time interval 300–480 ms after onset of S2 at Fz, Cz, and Pz. The ERPs for NoGo N2 and P3 were computed from a mean of 89.0 (SD = 11.8; range = 44–100) trials for the ADHD group, and 90.4 (SD = 10.8; range = 49–100) for the healthy controls. Baseline was adjusted to the mean voltage average 100 ms before stimulus presentation relative to S1 for the Cue P3 and late CNV, and relative to S2 for Go P3, NoGo N2, and NoGo P3.

### Procedure

All participants were interviewed by a senior neuropsychologist using the semi-structured Adult Interview [112], and filled out a Norwegian translation of the ASEBA [114] Adult Self-Report (ASR).

Prior to EEG recording and testing, all participants had been instructed to forgo taking any nicotine or alcohol, or psychoactive medication in line with physician recommendation, and starting no later than 6 pm the day before the assessment. On the morning of the EEG recording and testing, participants were evaluated related to testability and inquired about food intake, last night sleep, alcohol/nicotine use, and other drugs or conditions that could influence their cognitive performance. None had issues that prevented participation.

For all participants, the experimental session was conducted before noon, in a room without environmental distractions. It was individually administered in accordance with the general task requirements. The participants sat in a comfortable chair with a distance of 1.5 meters (4.92 feet) from a 17-inch computer screen. The EEG recording session started with two resting state sessions (eyes closed and eyes open). Next, participants were shown the different visual stimuli to be used in the cued Go/NoGo task and underwent a formal practice session prior to the actual task. The task instructions were given by a test technician who was present during the whole session.

### Statistical analysis

Statistical Product and Service Solutions for Windows [119] was used for the statistical analyses. The between-group difference in gender composition was analyzed with chi square. Comparison of group differences in other demographic (age, education, work/study status), psychometric (WAIS III; ASEBA ASR DSM and Problem scales), and cued Go/NoGo task performance (reaction time, reaction time variability, omission- and commission errors) data were calculated using independent samples t-test with group (ADHD vs. healthy control) as the between-subjects factor.

Based on the general hypothesis that all investigated ERP components would have reduced amplitudes in the ADHD compared with the healthy control group, power estimates were conducted based on previous studies indicating group differences in that direction. For the ERP components where the highest number of participants were needed, it was estimated that at least 27 participants per group would be required to achieve a power of .80 [115]. The NoGo N2 was added at a later phase and was not subjected to power analysis.

Group differences in ERP amplitudes and topographies were analyzed using multivariate analyses of variances (MANOVA). Multivariate analyses were performed instead of the more commonly used repeated measures (RM) ANOVA, as the RM ANOVA assumption of sphericity is often violated in ERP-data [120]. The analyses for the S1-related ERPs (cue P3 and late CNV) were defined by the within-subjects factors Condition (Relevant and Irrelevant cue), and Electrode (Cue P3: Cz and Pz; late CNV: Fz, Cz, and Pz). Analyses of the S2-related ERPs (Go P3 and NoGo N2 and P3) were defined by an within-subjects Electrode factor (Go P3: Cz and Pz; NoGo N2: Fz and Cz; NoGo P3: Fz and Cz and Pz). Group (ADHD vs. healthy control) was between-subjects factor in all analyses. Effect size was computed employing eta-squared (ƞ^2^) and partial ƞ^2^(ƞ_p_^2^). Significant interaction effects were decomposed using One-Way ANOVA. Tukey corrected p-values are reported in post hoc analyses. Pearson product-moment correlation coefficients (two-tailed test) were used to test within-group relationships of electrophysiological data (Pz for Cue P3;Cz for late CNV; Fz for NoGo N2; Cz for Go P3 and NoGo P3), and of performance scores on the cued Go/NoGo task, and ASEBA ASR DSM scale scores.

Alpha for statistical analyses was set at p ≤ .05, except for the correlation analyses where the more conservative significance level of ≤ .001 was chosen due to the large number of tests conducted.

## Results

### Behavioral data

Table 2 shows the group-wise performance data for the cued Go/NoGo task. Due to technical difficulties, behavioral data was missing for 1 ADHD patient and 1 healthy control. There were no significant differences between ADHD patients and healthy controls in reaction time (RT) (*t*(60) = -.14, *p =* .891,ƞ^2^ = .02) or reaction time variability (RT SD) (*t*(60) = 1.33, *p* = .188, ƞ^2^ = .34) to detected Go-signals. The ADHD group had significantly more Go trial omission errors (*t*(60) = 2.13, *p =* .037, ƞ^2^ = .27), and a tendency for more NoGo trial commission errors (*t*(60) = 1.88, *p =* .065, ƞ^2^ = .24) compared to the control group. Note that commission errors were seen in a minority of participants in both groups (ADHD: 28.1%; Control: 13.3%). For omission error, the incidence was high in both groups (ADHD: 90.6%; Control: 83.3%). None of the participants had more than 23% omission errors, and none had more than 4% commission errors.

**Table 2:** Behavioral results on the cued Go/NoGo task for ADHD patients (n = 32) and healthy controls (n = 30). Group means and standard deviations (SD) are reported.

	ADHD		Control	
	Mean	SD	Mean	SD
**Go signals**				
RT (ms)	422.4	69.2	424.9	77.5
RT SD (ms)	112.8	38.8	101.6	25.8
Omission errors	7.5	6.9	4.4	4.3
**NoGo signals**				
Commission errors	0.5	1.0	0.1	0.3

Notes. RT = reaction time; SD = standard deviation; ms = milliseconds; omission and commission errors = number of errors. There were missing behavioral data for 1 ADHD and 1 control participant.

### Electrophysiological data

The groups did not significantly differ in the number EEG trials used to calculate the Cue, Go, and NoGo ERPs (*p* values ranging from .361 to .885).The mean amplitudes of the Cue P3 and late CNV to the relevant S1 signal (S1 = Animal), P3 to the Go S2 signal (S2 = Animal), and N2 and P3 to the NoGo S2 signal (S2 = Plant or Human) are presented (Table 3).The mean amplitudes of Cue P3 and late CNV waves in the Irrelevant condition (S1 = Plant) are also shown.

**Table 3:** Mean ERP amplitudes (μV) for ADHD patients (n = 33) and healthy controls (n = 31). Group means, and standard deviations (SDs) and confidence intervals (CIs) are reported.

	ADHD			Control			ADHD			Control		
	Relevant trials						Irrelevant trials					
	Mean	SD	95% CI	Mean	SD	95% CI	Mean	SD	95% CI	Mean	SD	95% CI
**Cue P3 Go**												
Cz	0.2	1.0	0.23[-0.11–0.57]	0.4	1.1	0.39[-0.00–0.80]	-0.1	0.7	-0.13[-0.36–0.16]	0.0	1.1	-0.03[-0.35–0.45]
Pz	2.2	1.3	2.19 [1.79–2.70]	2.6	0.9	2.62[2.29–2.98]	0.9	0.8	0.94[0.61–1.21]	1.1	0.7	1.05[0.81–1.34]
**Late CNV**												
Fz	0.2	1.5	0.14[-0.37–0.71]	0.3	1.1	0.1[2–0.25–0.79]	0.3	0.9	0.31[-0.00–0.61]	0.5	1.1	0.46[0.14–0.96]
Cz	-1.5	1.3	-1.44[1.93–- 1.04]	-1.8	1.1	-1.76[-2.14–-1.37]	0.0	0.8	0.03[-0.24–0.31]	-0.1	0.8	-0.15[-0.41–0.21]
Pz	-1.5	0.9	-1.45[-1.81–-1.19]	-1.6	1.0	-1.52[-1.92–-1.18]	-0.2	0.7	-0.18[-0.48–0.02]	-0.5	0.8	-0.49[-0.79–-0.19]
**P3 Go**												
Cz	4.0	2.2	3.96[3.09–4.74]	5.6	2.5	5.43[4.63–6.48]						
Pz	5.7	2.0	5.89[5.02–6.46]	6.1	2.3	5.89[5.30–6.96]						
**N2 NoGo**												
Fz	-2.9	3.1	-2.90[-3.98–-1.76]	-3.1	3.1	-3.06[-4.27–-1.98]						
Cz	0.8	3.0	0.64[-0.28–1.82]	1.0	2.0	0.94[0.28–1.76]						
**P3 NoGo**												
Fz	1.6	2.3	1.46[0.75–2.38]	1.7	2.8	1.82[0.70–2.76]						
Cz	4.5	2.4	4.39[3.59–5.31]	6.5	3.2	6.34[5.30–7.64]						
Pz	3.3	2.0	3.19[2.56–3.98]	4.1	2.1	4.04[3.30–4.89]						

### ERPs to relevant and irrelevant cues

*Cue P3*: The 2 × 2 × 2 (Electrode x Condition x Group) MANOVA showed significant main effects of Condition (*F*(1, 62) = 82.95, *p* = .001, ƞ_p_^2^ = .57), and Electrode (*F*(1, 62) = 134.04, *p* = .001, ƞ_p_^2^ = .68), reflecting that the Cue P3 amplitude was larger for relevant compared to irrelevant cues across groups. There was no significant main effect of Group (*F*(1, 62) = 1.79, *p* = .185, ƞ_p_^2^ = .03), or interactions involving Group (Group x Electrode: *F*(1, 62) = 0.20, *p* = .654, Wilks’ Lambda = .997, ƞ_p_^2^ = .00); Group x Condition: (*F*(1, 62) = .42, *p* = .519, Wilks’ Lambda = .993, ƞ_p_^2^ = .01); Group x Electrode x Condition: (*F*(1, 62) = .61, *p* = .438, Wilks’ Lambda = .990, ƞ_p_^2^ = .01).

**Fig 2:**
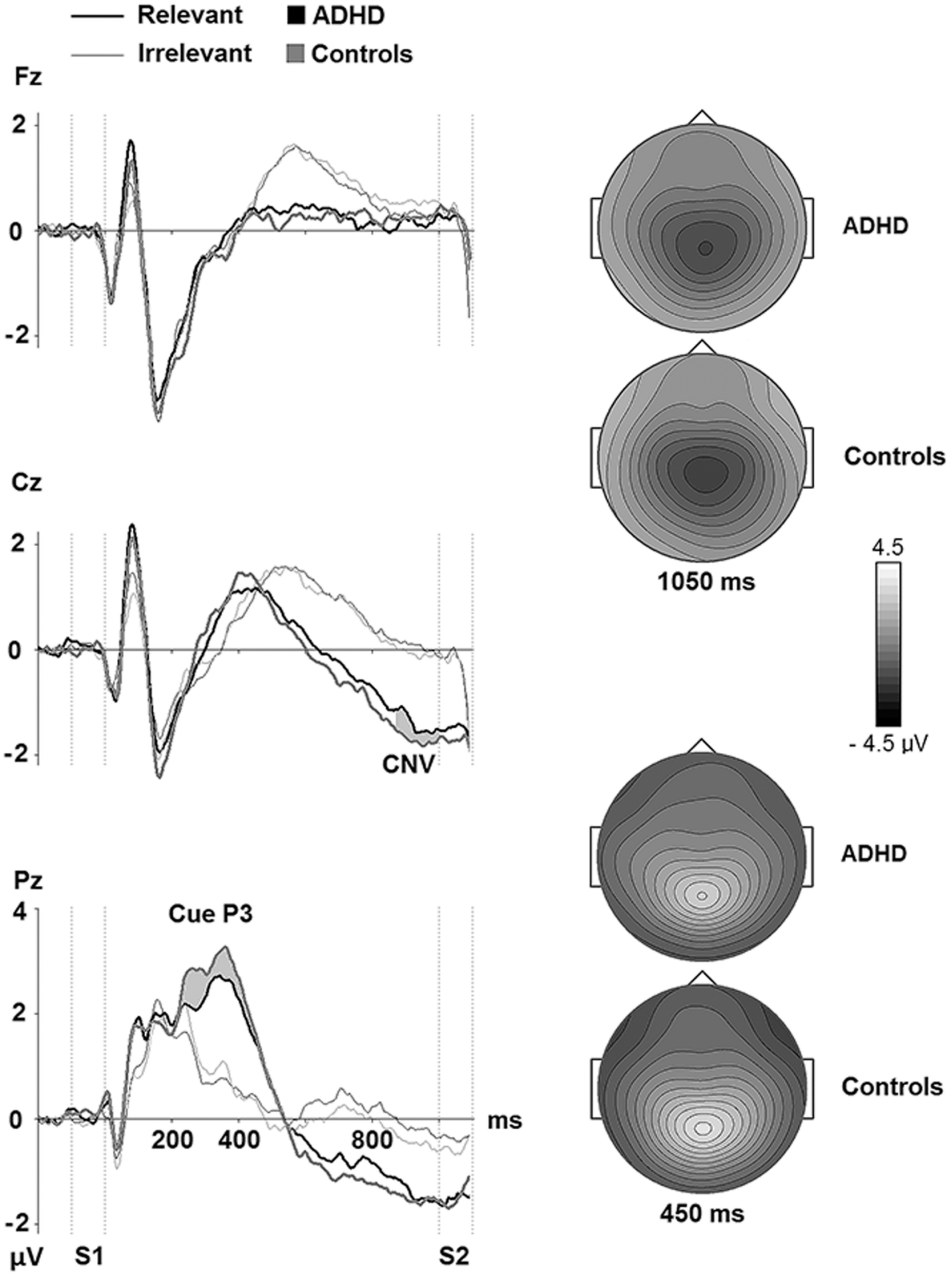
Grand average ERP waves over frontal, central, and parietal electrodes for the ADHD and the healthy control participants in Relevant (Animal = S1) and Irrelevant (Plant = S1) conditions in the cued Go/NoGo task. The vertical dotted lines indicate the duration of S1 and S2. The grey areas indicate the time intervals that the amplitudes of the Cue P3 (mean of the 300–550 ms interval after onset of S1) and the late CNV (mean of the last 100 ms before onset of S2) were extracted from. They are marked at the electrodes where the components had their maximum amplitudes. Topographic maps of the grand average maximum amplitudes at Cz for late CNV and Pz for Cue P3 are also shown.

*Late CNV*: The 3 x 2 x 2 (Electrode x Condition x Group) MANOVA showed significant main effects of Condition (*F*(1, 62) = 130.76, *p* = .001, ƞ_p_^2^ = .68), and Electrode (*F*(2, 61) = 49.10, *p* = .001, ƞ_p_^2^ = .62), which reflected that the late CNV amplitudes were generally larger for relevant than for irrelevant cues. The analyses showed no significant main effect of Group (*F*(1, 62) = 0.18, *p* = .674, ƞ_p_^2^ = .00), or interactions of Group and other factors (Group x Electrode: (*F*(2, 61) = 1.48, *p* = .236, Wilks’ Lambda = .954, ƞ_p_^2^ = .05); Group x Condition: (*F*(1, 62) = 0.02, *p* = .891, Wilks’ Lambda = 1.00, ƞ_p_^2^ = .00); Group x Electrode x Condition: (*F*(2, 61) = 1.11, *p* = .335, Wilks’ Lambda = .965, ƞ_p_^2^ = .04). See Fig 2.

### ERPs to Go and NoGo signals after relevant cues

**Fig 3:**
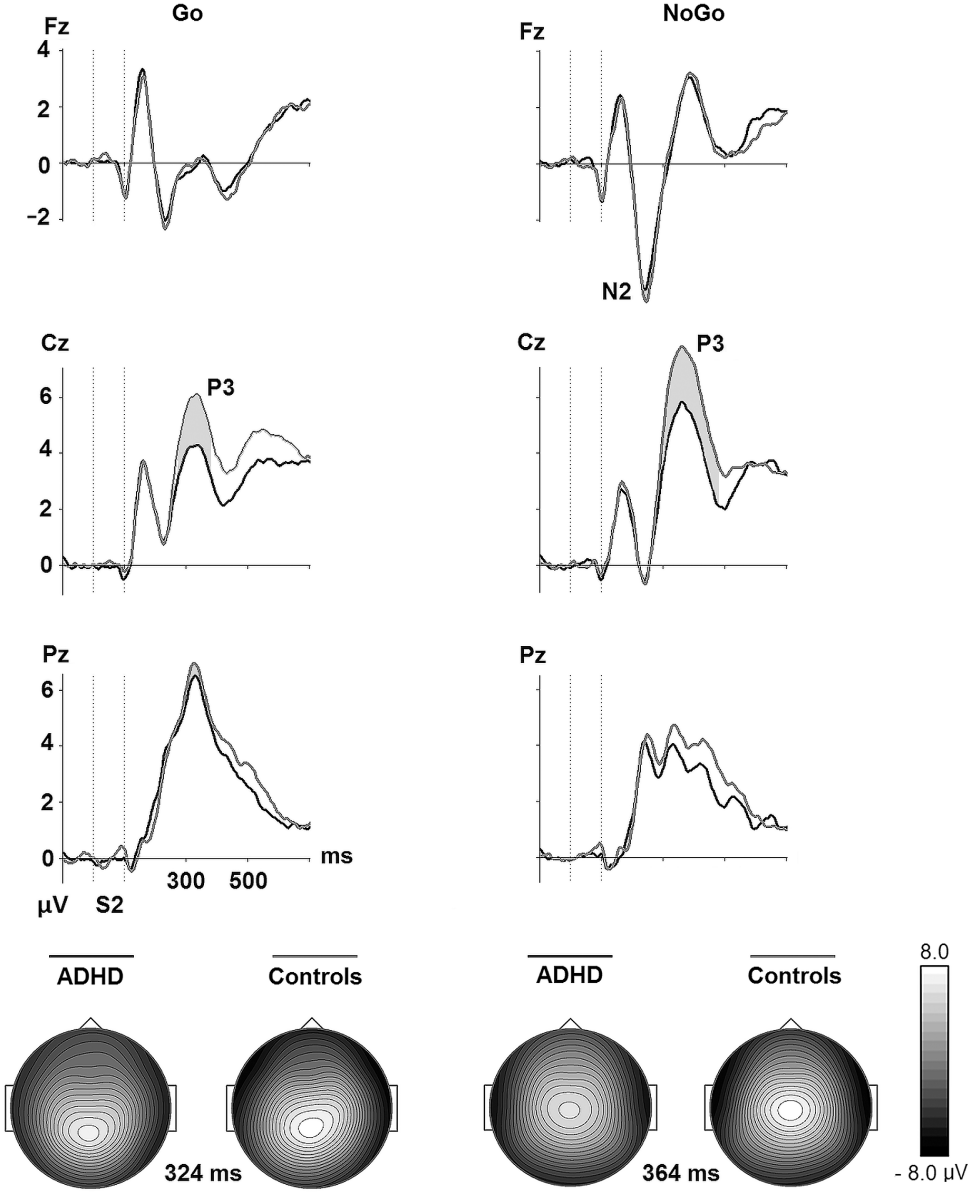
Grand average ERP waves over frontal, central, and parietal electrodes for the ADHD and the healthy control groups after S2 in Go (A—A) and NoGo (A—P) conditions in the cued Go/NoGo task. The grey areas indicate the time intervals that the mean amplitudes of the Go P3 (280–380 ms), the NoGo N2 (200–300 ms), and the NoGo P3 (300–480 ms) waves were extracted from after onset of S2. The grey areas are marked at the electrodes where the components are most clearly observed. Topographic maps of the grand average peak amplitudes at Cz for Go P3 and NoGo P3 are also shown.

*Go P3*: Fig 3 suggests that healthy controls had larger Go P3 at the central location compared to ADHD patients. Supporting this impression, the 2 x 2 (Electrode x Group) MANOVA revealed significant main effects of Group (*F*(1, 62) = 4.31, *p* = .042, ƞ_p_^2^ = .07), and Electrode (*F*(1, 62) = 15.42, *p* = .001, Wilks’ Lambda = .801, ƞ_p_^2^ = .20), that were modified by a marginally significant interaction of Group and Electrode (*F*(1, 62) = 3.97, *p* = .051, Wilks’ Lambda = .940, ƞ_p_^2^ = .06). Post hoc testing of the interaction effect showed that the ADHD patients had significantly smaller Go P3 at Cz relative to the controls (*F*(1, 62) = 7.25, *p* = .009, ƞ_p_^2^ = .11).

*NoGo N2*: The 2 x 2 (Electrode x Group) MANOVA revealed a significant main effect of Electrode for the NoGo N2 (*F*(1, 62) = 141.83, *p* = .001, Wilks’ Lambda = .304, ƞ_p_^2^ = .70), in that its amplitude was largest at Fz across groups. There was no significant main effect of group (*F*(1, 62) = 0.00, *p* = .995, ƞ_p_^2^ = .00), or interaction of Group and Electrode (*F*(1, 62) = 0.59, *p* = .444, Wilks’ Lambda = .991, ƞ_p_^2^ = .01). See Fig 3.

*NoGo P3*: The 3 x 2 (Electrode x Group) MANOVA for the NoGo P3 resulted in significant main effects of Electrode (*F*(2, 61) = 76.81, *p* = .001, Wilks’ Lambda = .284, ƞ_p_^2^ = .72), and Group (*F*(1, 62) = 5.02, *p* = .029, ƞ_p_^2^ = .08), that were modified by a significant Group by Electrode interaction (*F*(2, 61) = 5.30, *p* = .008, Wilks’ Lambda = .852, ƞ_p_^2^ = .15).

Post hoc tests revealed that the group differed significantly at Cz only (*F*(1, 62) = 8.19, *p* = .006, ƞ^2^ = .73). ƞ_p_^2^ = .12).

### Correlation analyses

#### Relationships between ERP components

Table 4 shows the within-group relations between the ERP components occurring after S1 (Cue P3 and late CNV) and components elicited to S2 (Go P3, NoGoN2and NoGo P3) in the ADHD group and the healthy control group. The within-group correlations between the Go P3 and the NoGo P3 are also shown. The general pattern for both groups was that the late CNV correlated negatively with both Go and NoGo P3, reflecting that the more negative amplitude of the late CNV the more positive amplitude of the Go P3 and NoGo P3. For the ADHD group the late CNV correlated negatively also with the NoGo N2. For the healthy control group the Cue P3 correlated positively with the NoGo N2, reflecting that the higher amplitude in the proactive Cue P3 the higher amplitude in the reactive NoGo P3.

**Table 4:** Within-group correlations between proactive (Cue P3, late CNV) and reactive (Go P3, NoGo N2, NoGo P3) ERPs in Go and NoGo trials for ADHD patients (n = 33) and healthy controls (n = 31).

	ADHD			Control		
	Go	NoGo		Go	NoGo	
	P3	N2	P3	P3	N2	P3
**Cue P3**	.26	-.18	.41	.03	.66*	.10
**Late CNV**	-.80*	-.36	-.72*	-.58*	-.09	-.71*

Notes. Selected electrodes for the components was Pz for Cue P3; Cz for late CNV; Fz for NoGo N2; and Cz for Go and NoGo P3;

* = p < .001.

#### Relations between task performance and ERPs in Go and NoGo trials

Table 5 shows the within-group relationships of behavioral performance to ERP amplitudes. ERP data extracted from the electrodes where the mean amplitudes reached their maximum voltage. Generally, the positive correlations involving task performance and late CNV reflected that diminished (less negative) amplitudes were related to worse performance scores. Negative correlations between performance measures and P3 components indexed that larger amplitudes were associated with better task performance. For the ADHD group, reaction time and its variability correlated positively with the late CNV, reflecting that the faster reaction time and less reaction time variability, the more negative amplitude of the late CNV. Further, the reaction time variability and omission error correlated negatively with Go and NoGo P3, reflecting that the less variability in reaction time and Go omission errors, the higher P3 amplitude in both Go and NoGo conditions. For the healthy control group, reaction time correlated negatively with NoGo P3, reflecting that the faster reaction time the larger NoGo P3 amplitude. Also, Go omission error correlated negatively with Go P3, meaning that the less Go omission errors the larger Go P3 amplitude. Omission error correlated positively with late CNV, reflecting that the less errors in the Go condition the larger CNV amplitude in the control group. Across groups, there were no significant correlations between NoGo trial commission errors and ERP parameters.

**Table 5:** Within-group correlations between behavioral performance and ERPs in a cued Go/NoGo task, and scores on ASEBA Adult Self-Report (ASR) DSM scale scores for ADHD patients (n = 32) and healthy controls (n = 30).

	ADHD					Control				
	Cue	Late	Go	NoGo		Cue	Late	Go	NoGo	
	P3	CNV	P3	N2	P3	P3	CNV	P3	N2	P3
**Cued Go/NoGo**										
RT_#_	-.03	.46*	-.37	.13	-.36	-.28	.30	-.31	.34	-.50*
RT SD_#_	-.37	.56*	-.57*	.02	-.56*	-.13	.42*	-.22	.06	-.38
OE_#_	-.23	.31	-.50*	.07	-.54*	.05	.50*	-.48*	-.17	-.38
CE_#_	-.05	-.07	-.11	-.04	-.21	.00	.12	-.15	-.15	-.21
**ASR DSM and Problem scales**										
ADHD_#_	-.06	.21	-.45*	-.36	-.29	.27	.28	-.01	-.33	-.12
Depression_#_	.12	-.07	-.08	-.32	.12	.08	.12	-.14	-.04	-.15
Anxiety_#_	.02	.00	-.12	-.16	.01	-.11	-.08	.07	.33	-.11
Antisocial_β_	-.17	.23	-.41	.05	-.31	.08	.29	-.15	.05	-.25

Notes. RT = reaction time; SD = standard deviation; OE = omission errors; CE = commission errors; Antisocial = Antisocial Personality Problems; Selected electrodes for the ERP components were Pz for Cue P3; Cz for late CNV; Fz for NoGoN2; and Cz for Go P3 and NoGoP3;

^#^ = missing data in1 ADHD patient and 1 healthy control;

^β^ = missing data in 5 ADHD patients and 5 controls;

* = p< .001.

#### Self-reported symptoms and their relationships to ERPs

Because there were significant differences between ADHD patients and healthy controls on the ADHD scale (*t*(60) = 11.04, *p =* .001), the Depression scale (*t*(60) = 9.41, *p =* .001), the Anxiety scale (*t*(60) = 6.41, *p =* .001), and the Antisocial Personality Problems scale (*t*(52) = 5.73, *p =* .001) of the ASEBA ASR DSM self-report (Table 1), we tested whether these symptom scores correlated with ERP responses.Table 5 shows the within-group relationships between electrophysiological responses and ASEBA ASR DSM results for both groups. In the ADHD group there were significant negative correlations between the ADHD scale and the GoP3 amplitudes, reflecting that the more self-reported problems on the ADHD scale, the smaller Go P3 amplitudes.

## Discussion

We investigated the behavioral and neurophysiological correlates of cognitive control processes during a visual cued Go/NoGo task in a cohort of treatment-naive adults with newly diagnosed ADHD. It was hypothesized that the ADHD group would show changes in ERP components indexing anticipatory attention and response preparation (Cue P3, late CNV), as well as ensuing processes associated with conflict monitoring and response control (Go P3, NoGo N2 and NoGo P3). We further predicted that indices of altered neural response patterns would be accompanied by reduced task performance. The hypotheses were partly confirmed, and we also observed some discrepancy between behavioral and electrophysiological indices of cognitive control. ADHD patients had more Go-signal omission errors than their healthy counterparts, but performed comparably to controls on reaction time measures. ERP responses did not uniformly differ from those of controls, but key measures of reactive attention and response control were diminished. Specifically, the Cue P3 and late CNV, as well as the NoGo N2 did not distinguish the groups, but the Go P3 and NoGo P3 amplitudes were significantly attenuated in the ADHD patients. Both groups showed moderate associations between ERPs and task performance. The general pattern was that reduced ERP amplitudes were moderately correlated to inferior performance, but not with depressed mood, anxiety, or antisocial personality problems. The results are discussed in relation to findings of previous studies, and how joint sampling of electrophysiological and behavioral measures provide complementary data for detailed analysis of cognitive control processes in adult ADHD.

### Performance on the cued Go/NoGo task

The ADHD patients performed similarly to healthy controls on measures of response speed and consistency, and the commission error rate only differed at a trend-level. In line with other adult ADHD studies [89, 93], the patients had more omission errors to Go signals than the controls. Although the patients had more attentional slips than controls, the behavioral results suggest that they adhered to the task instructions. Wiersema and coworkers (2009) also reported that the performance of an adult ADHD sample did not deviate from age-matched healthy control participants on a visual Go/NoGo task [121].This is in contrast to studies on ADHD children where deficient Go/NoGo performance is frequently observed [122]. Valko and colleagues [93], along with another ADHD study [61], found that adults performed better than children and interpreted this as developmental improvements of attention and inhibitory control in ADHD. However, adults with ADHD performed more poorly than age, sex and IQ-matched non-clinical controls with regard to reaction time, its variability, and target omission errors [93]. The tasks used differ in these studies and thus limits generalizing across studies. Especially, the commonly used cued CPT-OX task has been judged to be too easy for studying attentional control in an adult population, and the use of more demanding tasks has therefore been recommended [53].

The cued Go/NoGo paradigm employed in the present study was also used in an adult ADHD study by Muller and colleagues [118]. They reported a greater number of omission- and commission errors, and larger reaction time variability in the ADHD patients relative to the healthy controls. Inspection of the performance scores indicates that the healthy control group in the present study performed lower with respect to omission errors and reaction time variability than the controls in the Mueller et al. study. Differences in neuropsychological test performance has been associated with demographic factors such as age, sex, and education [123]. Control participants in the present study were matched to the patients on the variables age, sex, and years of education, whereas the participants in the Mueller et al. study were reported to have been matched on age and sex only. The difference between the two studies regarding performance scores of the control groups may relate to demographic variables.

Reaction time instability [124], together with an increased rate of Go signal omission errors [34], and/or commission errors to NoGo stimuli, are commonly observed in both children and adults with ADHD performing CPTs [11, 33, 94, 125, 126]. Such findings are typically interpreted as indices of inattention and deficits in inhibitory control, respectively. This pattern of impaired performance may not be observed when the demand for sustained focused attention is reduced by a break in the recording session as employed in the present Go/NoGo task. The inclusion of a break may make ADHD patients less likely to commit errors that are due to reduced sustained vigilance.

Thus, behavioral measures in the present task may not be as sensitive to the inattention and inhibitory control deficits often reported in patients with ADHD doing typical CPTs without the midway break [103]. However, despite performing at the same level as healthy controls on most behavioral parameters, the amplitudes of the ADHD group’s reactive electrophysiological responses were altered in both Go and NoGo trials.

### Electrophysiological responses in the cued Go/NoGo task

The Cue P3 and late CNV were investigated as electrophysiological indices of processes related to relevance evaluation, sustained anticipation and motor preparation for potential Go signals (S2) succeeding the cues (S1). Inspection of the Cue P3 and the late CNV at centro-parietal sites (Fig 2) suggests comparable ERP measures of preparatory processing with neither the amplitude of the Cue P3 nor the late CNV differing between groups. Fig 2 also illustrates that both groups distinguished between goal-relevant and goal-irrelevant cues, as cues in the Irrelevant trials evoked less positive- and negative-going responses in the time windows and locations for the Cue P3 and late CNV than cues signaling potential Go stimuli. The Cue P3 and late CNV data indicate that neural processes related to allocation of attentional resources to the relevance evaluation of the cue and to the preparation for upcoming stimuli did not differ between ADHD and control participants. Compared to recent literature, these results differed from some studies which found that boys (8–16 years) with ADHD and their non-affected siblings had reduced Cue P3 and CNV in CPTs with a high attentional load [127], whereas another study [49] showed that boys in the same age range had attenuated Cue P3, but had CNVs that did not differ from typically developing controls performing a flanker cued-CPT task. However, no Cue P3 group differences, but attenuated CNV were found when women with ADHD were compared to healthy control women [54]. Moreover, in a study using a standard CPT task, the authors found that both children and adults with ADHD had smaller CNV than age-matched non-clinical controls [93]. McLoughlin and colleagues (2010) reported that adults with ADHD performing a standard CPT task had attenuated Cue P3 and a trend for reduced CNV amplitudes compared to non-clinical controls. The patients also had reduced CNV in a flanker version of the task [89]. A study of children with ADHD who were followed with repeated electrophysiological assessments of the Cue P3 and the CNV into young adulthood showed that only the CNV remained reduced [44]. These authors found that the developmental trajectory of the CNV in the ADHD group paralleled the rising curve of healthy controls, but at a lower level. They concluded that the deficits in ADHD cannot be explained by developmental lag which imply a brain maturation normalization, and argued that attenuated or deviant development would better explain their findings [44]. Adult males with ADHD who showed deficiency in response control measures, indexed by greater response time variability and a attenuated NoGo P3, did not have attenuated CNV [53]. The results of the present study indicate, along with other studies, that a diminished late CNV to cues is not an invariant feature of adult ADHD.

The hypothesized reduction of P3 amplitudes was found for the ADHD group with both the Go P3 and the NoGo P3 amplitudes decreased relative to the control group. Attenuated P3 ERPs have been reported in children and adolescents [46, 60, 127], as well as adults [94, 95, 128, 129] with ADHD, and are commonly thought to reflect a limitation in attentional resources allocated to tasks demanding effort, timing, and inhibitory control. Decreased P3 Go amplitude may reflect altered neural processes underlying the attentional lapses indexed by increased omission errors in the ADHD group. This interpretation is supported by the finding that decreased Go P3 was related to a higher rate of omission errors. A review by Szuromi and colleagues did not report a consistent relationship between task performance and the target-related P3, and viewed this as support for the idea that reduced P3 amplitude may be an independent electrophysiological indicator of attentional disturbances in adult ADHD [45]. Even if a diminished P3 is not a disorder-specific phenomenon [23], it may provide significant information about the functional problems in ADHD. In the current study, the Go P3differed predominantly between ADHD patients and controls at Cz, and was related to the DSM ADHD score in that reduced amplitude was associated with more self-reported ADHD problems in the patient group. The Go P3 maximal at Cz has been suggested to reflect frontal attention mechanisms engaged to stimulus processing [42, 130], or monitoring of attention[117]. Most studies of ADHD do not distinguish between different Go P3 components. Different task paradigms may evoke Go P3s that partly differ in topographical distribution and underlying attentional processes, which could be one explanation for the inconsistent findings regarding the Go P3 in ADHD studies. In future studies, a differentiation between central and parietal P3s may contribute to increase the knowledge base of cognitive control processes in ADHD.

The low rate of commission errors accompanied by the diminished NoGo P3 in the adult ADHD group, and the lack of significant correlation between them, might fit with the view that NoGo P3 is not an index of the inhibitory process itself, but alternatively reflects evaluation of the inhibitory process or its outcome [39]. In case of the latter, the attenuated NoGo P3 would be an electrophysiological marker of reduced resources allocated to the evaluation of the action control processes. Decreased NoGo P3 amplitudes were associated to more Go signal omission errors and increased reaction time variability for the patients, when the increased reaction time was related to decreased NoGo P3 amplitudes in the control group. These results indicate that the magnitude of the NoGo P3 is related to aspects of behavioral performance across groups that extend beyond those measured in the NoGo trials of the task. The strong associations between the Go P3 and NoGo P3 in both groups indicate a possible common denominator with respect to function, such as active facilitation of the task-relevant behavioral reaction, i.e., implementation of an expected and prepared response to targets in the case of Go P3, and implementation of an alternative to the Go response (i.e., a non-response) in the case of NoGo P3.Of note, S2 in the Irrelevant conditions did not elicit a NoGo P3 (Fig 2) in either group, indicating an ability to not release attentional resources to stimuli that have been defined as task-irrelevant by the preceding cue.

The magnitude of the N2 preceding the P3 in NoGo trials did not distinguish patients from controls. The results are in line with findings in a study of male adults with ADHD who showed no change in NoGo N2compared to age-matched healthy controls [129], and a study of ADHD children who like their healthy controls had increased N2 in NoGo compared to Go trials [23]. Increased N2 in NoGo trials has been associated with the perceptual and cognitive component of the inhibition process [78, 83], or conflict monitoring rather than the motor response suppression itself [39]. In line with this understanding of NoGo N2, our ADHD sample did not perceive and monitor response conflict differently from controls as indicated by the magnitude and scalp topography of the NoGo N2. Moreover, N2 showed a differential sensitivity to NoGo versus Go trials in both groups (Fig 3). Notably, the NoGo N2 had no significant relationship to the rate of commission errors or other behavioral task parameters.

As in the present study, electrophysiological and behavioral parameters often show negligible or only modest correlations [45, 131]. This may be because the measures are sensitive to partly different aspects of information processing, providing different windows into brain function. There are several reasons why group differences in ERPs are accompanied by more limited differences in behavioral performance. One possibility is a difference in task strategy. An alternative interpretation is that altered ERPs represent deficits in one or more processes that behavioral measures are not sensitive enough to detect. Reduced amplitudes of late cognitive ERP responses such as the Go and NoGo P3 may reflect a limited availability, or expenditure of available attentional resources to stimulus- or performance outcome evaluation. The tendency for weaker task performance with smaller amplitudes of ERPs indexing cognitive control may support this contention.

The magnitude of the ERP components were not significantly associated with self-reported mood, anxiety, or antisocial behavior. The results indicate that some elevation of emotional distress and social problems in the ADHD group (Table 1) did not modulate the electrophysiological markers of preparatory and reactive cognitive control processes.

### Limitations and strengths of the study

The present patient cohort was unique in the sense that patients were previously undiagnosed and had no history of central stimulation medication. Long-term effects of medications on brain neurobiology have been reported [132–134], and even temporary cessation of medication prior to participation in research may influence behavioral [135] and electrophysiological measures [136], confounding the interpretation of results.

The sizes of the study samples were modest. Thus, the suggestive findings reported here await replication in future studies with larger cohorts. The broad age range of the samples may hide potential different aging trajectories in the patient and the control group. Future studies should aim to include larger samples covering the full length of the adult life span in order to address this issue as well as potential gender differences.

## Summary and Conclusions

Our cohort of treatment-naive adults with ADHD had similar performance and ERP metrics to controls. Moreover, the topographic distributions and morphologies of the waveforms elicited by S1 and S2 were similar across groups both in the Go, NoGo, and Irrelevant task conditions. At the physiological level, this indicates that the neural generators of the waveforms operated in an analogous manner across groups. At the cognitive level, the results suggest that the two groups assigned task relevance to the stimuli in similar ways.

The most striking group difference was the attenuation of Go P3 and NoGo P3amplitudes in the ADHD group. The attenuated P3s were accompanied by a higher rate of Go-signal omission errors. We suggest that the P3 amplitude reductions may be related to deficits in effortful allocation of attentional resources to task stimuli demanding reactive response control. The association of NoGo P3 amplitude to prolonged Go signal response speed only seen in the control group supports this interpretation. The electrophysiological results highlight specific information processing deficits in the adult ADHD population in that late reactive-, but not proactive cognitive processes were altered.
