# Correction: ERP Correlates of Proactive and Reactive Cognitive Control in Treatment-Naïve Adult ADHD

**DOI:** 10.1371/journal.pone.0163404

**Published:** 2016-09-15

**Authors:** Venke Arntsberg Grane, Jan Ferenc Brunner, Tor Endestad, Ida Emilia S. Aasen, Juri Kropotov, Robert Thomas Knight, Anne-Kristin Solbakk

There are values missing from the SD column in Table 1. Please see the corrected Table 1 here.

**Table 1:** Demographics, IQ scores on WAIS-III, and T-scores on the ASEBA Adult self-Report (ASR) DSM scales for ADHD patients (n = 33) and healthy controls (n = 31).

	ADHD		Control		
	Mean	SD	Mean	SD	p
**Age** (years)	31.3	9.6	31.9	10.0	.788
**Education** (years)	10.8	1.6	11.3	2.2	.057
**Gender** (M/F)	14/19		14/17		.825
**Work or study** (number/%)	19/58		31/100		.001
**WAIS-III** (IQ points)					
FSIQ	93.7	13.0	98.9	10.3	.086
VCI _†_	93.3	13.6	95.6	11.3	.470
POI _†_	104.4	13.3	108.9	14.3	.200
WMI _†_	87.9	12.7	93.9	14.2	.081
PSI _†_	90.4	11.6	100.3	11.8	.001
**ASR DSM and Problem scales** (t-scores)					
ADHD _#_	74.7	10.7	52.2	3.2	.001
Depression _#_	67.1	9.2	50.9	2.1	.001
Anxiety _#_	59.2	7.4	50.4	0.9	.001
Antisocial _β_	63.1	9.4	52.0	3.4	.001
Alcohol use _π_	56.9	7.0	54.8	4.7	.220

Notes. Education = total years of schooling completed; Work or study = working or studying during the last 6 months; FSIQ = Full Scale Intelligence Quotient; VCI = Verbal Comprehension Index; POI = Perceptual Organization Index; WMI = Working Memory Index; PSI = Processing Speed Index; Alcohol use = days being drunk during the last 6 months;

^†^ = missing data in 1 ADHD patient;

^#^ = missing data in 1 ADHD patient and 1 control;

^β^ = missing data in 5 ADHD patients and 5 controls;

^π^ = missing data in 8 ADHD patients and 7 controls.
